# Qualitative Parameters of the Colonic Flora in Patients with HNF1A-MODY Are Different from Those Observed in Type 2 Diabetes Mellitus

**DOI:** 10.1155/2016/3876764

**Published:** 2016-10-11

**Authors:** Sandra Mrozinska, Piotr Radkowski, Tomasz Gosiewski, Magdalena Szopa, Malgorzata Bulanda, Agnieszka H. Ludwig-Galezowska, Iwona Morawska, Agnieszka Sroka-Oleksiak, Bartlomiej Matejko, Przemyslaw Kapusta, Dominika Salamon, Maciej T. Malecki, Pawel Wolkow, Tomasz Klupa

**Affiliations:** ^1^Department of Metabolic Diseases, Jagiellonian University Medical College, 15 Kopernika Street, 31-501 Kraków, Poland; ^2^University Hospital, 36 Kopernika Street, 31-501 Kraków, Poland; ^3^Center for Medical Genomics OMICRON, Jagiellonian University Medical College, 7c Kopernika Street, 31-034 Kraków, Poland; ^4^Department of Microbiology, Jagiellonian University Medical College, 18 Czysta Street, 31-121 Kraków, Poland

## Abstract

*Background*. Type 2 diabetes mellitus (T2DM) is determined by genetic and environmental factors. There have been many studies on the relationship between the composition of the gastrointestinal bacterial flora, T2DM, and obesity. There are no data, however, on the gut microbiome structure in monogenic forms of the disease including Maturity Onset Diabetes of the Young (MODY).* Methods*. The aim of the investigation was to compare the qualitative parameters of the colonic flora in patients with HNF1A*-*MODY and T2DM and healthy individuals. 16S sequencing of bacterial DNA isolated from the collected fecal samples using the MiSeq platform was performed.* Results*. There were significant between-group differences in the bacterial profile. At the phylum level, the amount of Proteobacteria was higher (*p* = 0.0006) and the amount of Bacteroidetes was lower (*p* = 0.0005) in T2DM group in comparison to the control group. In HNF1A-MODY group, the frequency of Bacteroidetes was lower than in the control group (*p* = 0.0143). At the order level, Turicibacterales was more abundant in HNF1A-MODY group than in T2DM group.* Conclusions*. It appears that there are differences in the gut microbiome composition between patients with HNF1A-MODY and type 2 diabetes. Further investigation on this matter should be conducted.

## 1. Introduction

Diabetes mellitus is a chronic disease that has become a major health and economic problem nowadays. 387 million people in the world suffer from diabetes and it is forecast that the number will be 205 million more in 2035 [1]. The health expenditure in the United States reached 612 billion American dollars in 2014 [1]. The state expenses incurred on the disease include not only medications, hospital inpatient care, and medical visits but also indirect costs like absenteeism, reduced productivity, and inability to work [2]. Because of that, more attention is paid to prophylaxis of obesity as well as to optimization and individualization of diabetes treatment [3].

Type 2 diabetes mellitus (T2DM) is determined by genetic and environmental causes. The risk factors contributing to the disease are, inter alia, age, body mass index, physical inactivity, and sedentary lifestyle [4, 5]. As far as genetic factors predisposing to T2DM are concerned, genetic variants modifying the risk of development of the disease have been identified; however, altogether, they explain only a small proportion of the genetic background of T2DM [6, 7]. Among possible causes of “missing heritability” gene, environment interactions are mentioned [8]. In recent years, there have been many studies on the relationship between the composition of the gastrointestinal bacterial flora, T2DM, and obesity [9–11]. These analyses indicate that the etiology of the various forms of diabetes, especially those associated with obesity and insulin resistance, such as T2DM, may be related to intestinal microflora [9, 10]. On the other hand, monogenic forms of the disease, like Maturity Onset Diabetes of the Young (MODY), are determined mostly by genetic factors, with minor effect of the environment. HNF1A-MODY is one of the most common forms of subtypes of MODY [12, 13]. It is caused by mutation in HNF1A (Hepatic Nuclear Factor 1 Alpha) gene. HNF1A-MODY is characterized by progressive hyperglycemia, with the onset of diabetes in youth. If not treated properly, it can lead to micro- and macrovascular complications [14]. Unlike in T2DM, HNF1A-MODY patients are usually slim [15]. The differences in the gut microbiota between patients with T2DM and HNF1A-MODY in relation to healthy individuals could be a new argument for an influence of microbiota as an environmental risk factor for some forms of diabetes. The gut microbiota perform multiple functions, produce vitamins, inhibit the growth of potential pathogenic bacteria, and lower the cholesterol level in blood [16]. The bacterial metabolite indole can modulate the secretion of glucagon-like peptide-1 (GLP-1) by L-cells [17]. It is hypothesized that remission of diabetes after bariatric surgery is connected with neurohormonal signaling or changes in the ecosystem of microbiota of the gastrointestinal tract [18, 19]. The intestinal microbiota play a role in the circulation of bile acids [20]. It is suggested that the increased levels of bile acids after bariatric operation stimulate GLP-1 secretion and lead to improvement in glucose metabolism [21]. The nuclear farnesoid X receptor (FXR) is also the molecular target for metabolic condition after bariatric surgery. The new approach is to assess the bacterial flora through the use of the noncultivated methods allowed for identifying bacteria that are difficult to culture [22]. These include targeted gene sequencing, genome sequencing, and shotgun metagenomic sequencing [23].

*The Aim*. The aim of the investigation was to compare the qualitative parameters of the colonic flora in patients with HNF1A*-*MODY diabetes, matched controls, and patients with T2DM in a single stool sample.

## 2. Materials and Methods

The study was designed in collaboration with the Department of Metabolic Diseases, Jagiellonian University Medical College, Kraków, Poland; University Hospital, Kraków, Poland; and Center for Medical Genomics OMICRON and Department of Microbiology, Jagiellonian University Medical College, Kraków, Poland. The research was performed in the years of 2013–2015. The project has obtained the consent of the Ethics Committee of the Jagiellonian University number KBET/272/B/2013.

Participants were the patients of the Department of Metabolic Diseases and volunteers, all of whom consented to participate in the research. We collected stool samples from ten patients with HNF1A-MODY and twenty-three patients with T2DM. All participants declared not using antibiotics 4 weeks prior to stool sample delivery and they denied having met the exclusion criteria (see the following list).

*Exclusion Criteria*

 Patients who did not agree to participate in the study and who withdrew during the investigation
 Patients below 18 years of age and over 65 years of age
 Patients taking antibiotics for up to 30 days before giving the sample of stool
 Patients using probiotics
 Patients with confirmed infection of the gastrointestinal tract
 Patients with chronic inflammatory bowel disease of unknown etiology
 Patients with active cancer (especially of the gastrointestinal tract)
 Patients with immunodeficiency
 Patients with features of liver damage (with the exception of nonalcoholic fatty liver transaminase levels less than three times the upper limit of the normal level)

In most patients, blinded continuous glucose monitoring (CGM, iPro2, Medtronic) was implemented. In addition, we utilized results of stool sample analysis of healthy individuals done previously (project supported by the National Science Centre in Poland, number DEC-2011/03/D/NZ5/00551). One control group of healthy individuals was matched with MODY patients with respect to age and BMI (body mass index), and the second one was matched to T2DM patients with respect to age.

The bacterial DNA was isolated using Genomic Mini AX Stool Spin (A&A Biotechnology) modified to include enzymatic treatment (lysozyme, lysostaphin, and lyticase) and bead-beating step. Libraries were prepared according to Illumina 16S Metagenomic Sequencing Library Preparation protocol (https://support.illumina.com/downloads/16s_metagenomic_sequencing_library_preparation.html). In short, universal external primers were used to amplify regions V3 and V4 of 16S rDNA. After the polymerase chain reaction (PCR) clean-up, samples were indexed, cleaned, and pooled. 10 pM libraries with 10% PhiX Spike-In were sequenced on Illumina MiSeq using V3 sequencing kit (300 bp paired end reads). Sample quality was evaluated using FastQC tool. PCR primers and sequencing adapters were trimmed using cutadapt. Resulting short reads were joined on overlapping regions using fastq-join tool from ea-utils package. Both joined and forward unjoined reads were used for further analysis. Reads with base quality lower than 20 were filtered out. OTUs (operational taxonomic units) were picked using open-reference protocol. In the first step, closed reference OTU picking is done against Green Genes 13.08 reference database. Subsequent reads that failed to hit the reference database were filtered out and used to perform de novo OTU picking. Reads were clustered using UCLUST. Taxonomy assignments were performed with PyNAST. Singletons OTUs were removed before further analyses. Relative OTU abundance was calculated using QIIME (http://qiime.org/). To estimate alpha diversity Chao 1, the observed OTUs and Phylogenetic Distance metrics were calculated. Both weighted and unweighted UniFrac distances were calculated to analyze beta diversity. Results were transformed using PCoA and visualized with Emperor. Frequency of OTU across sample groups was compared using nonparametric ANOVA (Kruskal-Wallis test) and Mann-Whitney *U* test. Post hoc tests using Statistica were performed if there were significant differences between groups. To correlate clinical parameters with relative OTU abundance, Spearman's Rho test was applied. *p* values were estimated using *Z* Fisher transform test. To compare the clinical parameters, the adequate parametric (*t*-test) and nonparametric (Mann-Whitney *U* test, Kruskal-Wallis test) tests were used.

As diet is concerned at disease diagnosis, all MODY and T2DM patients were educated to follow the diet recommended in diabetes with 40–50% of calories coming from carbohydrates, 20–30% from fat, and 20% from protein. The current diet knowledge and adherence were verified with the questionnaire.

## 3. Results

### 3.1. The Groups' Characteristics

**Figure 1:**
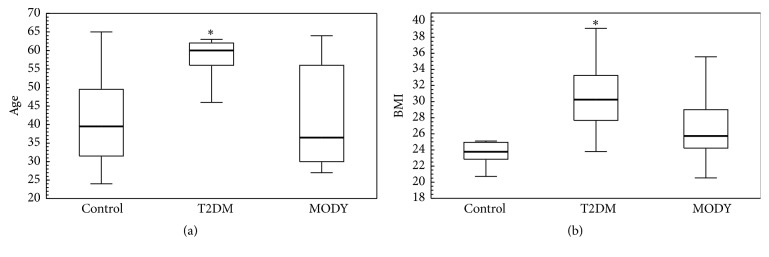
Comparison of age and BMI between groups. To reveal differences in age and BMI, the Kruskal-Wallis test was performed. Data presented as median (first–third quartiles). BMI: body mass index; MODY: Maturity Onset Diabetes of the Young; T2DM: type 2 diabetes mellitus. (a) Median age in the first control group was 39.5 (31.5–49.5) years, in HNF1A-MODY was 36.5 (30–56) years, and in T2DM was 60 (56–62) years. There was no difference in age (*p* = 1.0000) between HNF1A-MODY and the first control group. The T2DM group was older than the first control group (*p* = 0.0006) and HNF1A-MODY group (*p* = 0.0133). (b) Median BMI in the first control group was 23.77 (22.85–24.95) kg/m^2^, in HNF1A-MODY was 25.74 (24.22–29 kg/m^2^), and in T2DM was 30.25 (27.68–33.25) kg/m^2^. There was no difference with respect to BMI (*p* = 0.2600) between HNF1A-MODY and the first control group. The T2DM group was characterized by higher BMI than control group (*p* = 0.0000) and HNF1A-MODY group (*p* = 0.0290). *∗* indicates that there was a significant difference between this group and control group.

There were 23 T2DM patients (13 women and 10 men) and 10 HNF1A-MODY individuals (5 women and 5 men) included in the study. The median age in the T2DM group was 60 (56–62) years and in the HNF1A-MODY group was 36.5 (30–56), *p* = 0.0133. The mean duration of diabetes in T2DM group was 4.46 ± 2.95 years versus 19.1 ± 13.37 years, *p* < 0.0001. The median HbA1c (glycosylated haemoglobin) level in T2DM group was 8.12% (7.11%–8.78%) versus 7.205% (5.4%–7.86%), *p* = 0.0479, respectively. The first control group, matched for age (*p* = 1.0) and BMI (*p* = 0.26) with HNF1A-MODY group, consisted of 16 healthy individuals (11 women and 5 men) with median age of 39.5 (31.5–49.5) years and median BMI of 23.77 (22.85–24.95) kg/m^2^. The median BMI in HNF1A-MODY group was 25.74 (24.22–29) kg/m^2^ and in T2DM group was 30.25 (27.68–33.25) kg/m^2^, *p* = 0.0290. The T2DM group differed in age (*p* = 0.0006) and BMI (*p* < 0.0001) in comparison to the first control group (Figure 1). The second control group consisted of 10 people (7 women and 3 men). The median BMI in the second control group was 25.57 (24.32–27.38) kg/m^2^, and the median age was 56 (53.5–58) years. There was no difference in age between this control group and T2DM group (*p* = 0.3059), but the groups differ in BMI (*p* = 0.0012).

Based on the questionnaire data, we found no major differences in the dietary profile between T2DM and MODY patients. The questionnaire included four possible answers regarding following the prescribed diet: always, most of the time, rarely, or never. The majority (16 out of 23 patients with T2DM and 8 out of 10 individuals with MODY) declared following always or most of the time the diet recommended in diabetes with respect to the proportion of calories coming from carbohydrates, fat, and protein and with respect to food quality. Unfortunately, we had no reliable data concerning diet in control group.

The patients' treatment included metformin, sulfonylurea, acarbose, and insulin (Supplementary Material available online at http://dx.doi.org/10.1155/2016/3876764).

### 3.2. 16S rRNA Sequencing

The analysis of the samples by sequencing obtained an average of 180353.52 paired reads per sample, with median 140780 and standard deviation 152430.2. The best sample contained 843416 read pairs, while the worst contained 40878. After preprocessing (removing adapters, PCR primers, filtration of low quality bases, pair joining, and removal of chimeric reads), we received an average of 115782.34 readings per sample, with median 87994 and standard deviation 104992.4. The best sample contained 579694 readings, while the worst contained 16371. OTU picking resulted in 30736 OTUs. Statistical analysis included 639 OTUs, those with abundance of at least 0.01%. The bacterial composition was analyzed at the phylum, class, order, family, and genus level.

### 3.3. The Bacteria Profile

**Figure 2:**
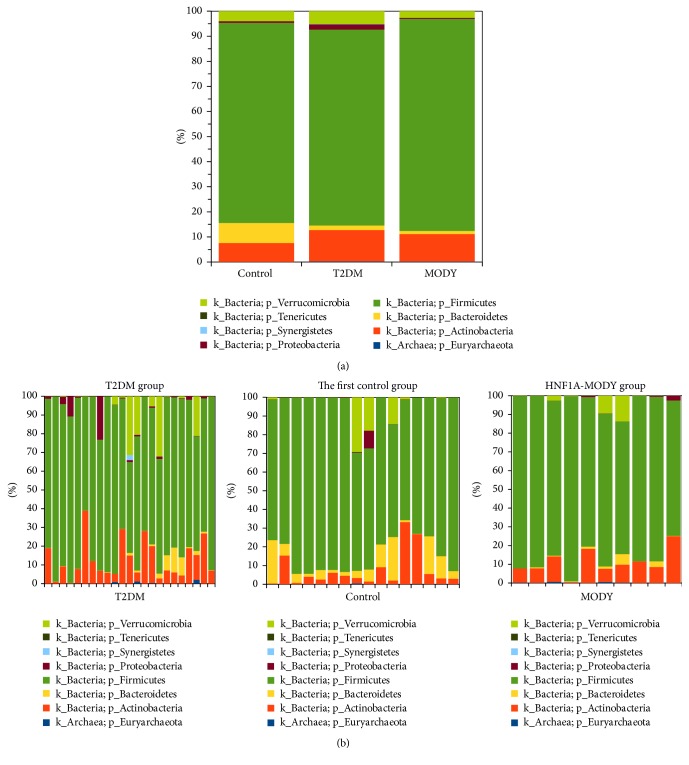
Comparison of frequencies of OTUs at the phylum level: (a) between groups (the first control group, HNF1A-MODY group, and T2DM group) and (b) between samples.

**Figure 3:**
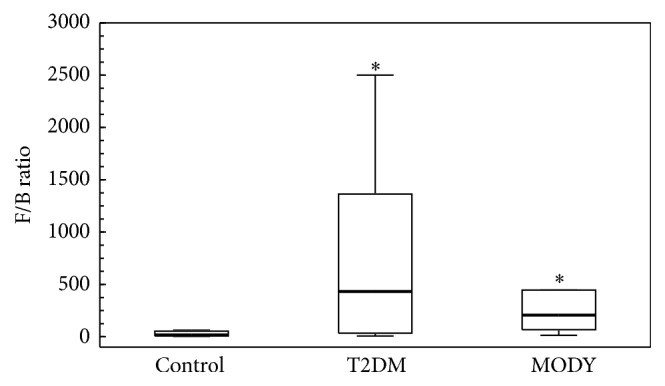
Box plots presenting the Firmicutes/Bacteroidetes ratio comparison between groups. The Firmicutes/Bacteroidetes (F/B) ratio was significantly higher in both T2DM (*p* = 0.0005) and HNF1A-MODY (*p* = 0.0113) groups than in the control group. There was no difference between T2DM and HNF1A-MODY groups. To show differences in F/B ratio, the Kruskal-Wallis test was performed. *∗* indicates that there was a significant difference between this group and control group.

The bacteria and archaea profiles for the first control, HNF1A-MODY, and T2DM at the phylum level were as follows: Euryarchaeota 0.12% versus 0.11% versus 0.13%; Actinobacteria 9.53% versus 9.31% versus 11.70; Bacteroidetes 6.39% versus 1.09% versus 1.30%; Firmicutes 77.02% versus 87.36% versus 80.09%; Proteobacteria 0.36% versus 0.35% versus 2.82%; Synergistetes 0.00% versus 0.00% versus 0.10%; Tenericutes 0.05% versus 0.005% versus 0.001%; Verrucomicrobia 6.52% versus 1.78% versus 3.85% (Figure 2). There were significant differences between T2DM and the first control groups at the phylum level (Kruskal-Wallis test with a False Discovery Rate (FDR) correction). In T2DM group, in comparison to the first control group, the amount of Proteobacteria was higher (*p* = 0.0006) and the amount of Bacteroidetes was lower (*p* = 0.0005). In HNF1A-MODY group, the frequency of Bacteroidetes was lower than in the control group (*p* = 0.0143). There were no significant differences between T2DM and HNF1A-MODY groups at the phylum level. The Firmicutes/Bacteroidetes* (F/B)* ratio was significantly higher in both T2DM and HNF1A-MODY groups than in the first control group (Figure 3). The Firmicutes/Bacteroidetes ratio was correlated positively with the HbA1c level in HNF1A-MODY group; we did not observe such phenomenon in T2DM.

There were significant differences at the class level: the proportion of Flavobacteria and Bacteroidia was higher in the first control group than in T2DM and HNF1A-MODY groups. The amount of Gammaproteobacteria was higher in T2DM group than in the first control group (*p* = 0.0002). The amount of Erysipelotrichi was higher in HNF1A-MODY group than in the first control group and T2DM group (supplementary data).

There were significant differences at the order level: Flavobacteriales and Bacteroidales were higher in the first control group than in HNF1A-MODY and T2DM groups. Actinomycetales was lower in the first control group than in HNF1A-MODY and T2DM groups. Turicibacterales was higher in the first control group than in T2DM group (*p* = 0.0073), and also Turicibacterales was higher in HNF1A-MODY group than in T2DM group. Enterobacteriales was higher in T2DM group than in the first control group. Erysipelotrichales was higher in HNF1A-MODY group than in the first control group and in T2DM group (supplementary data).

There were significant differences at the family level: Flavobacteriaceae and Bacteroidaceae were higher and Promicromonosporaceae and* the other family of *Actinomycetales were lower in the first control group than in both T2DM and HNF1A-MODY groups. Enterobacteriaceae was higher and Porphyromonadaceae was lower in T2DM group than in the first control group (*p* = 0.004). The frequency of Clostridiaceae and Turicibacteraceae was lower in T2DM group than in the first control group and HNF1A-MODY group. The amount of Erysipelotrichaceae was higher in HNF1A-MODY group than in the first control group and T2DM group (supplementary data).

There were significant differences at the genus level:* Bacteroides* and the* unnamed genus of* Flavobacteriaceae were higher and* Cellulosimicrobium* and the* other genus of the other family of *Actinomycetales were lower in the first control group than in T2DM and HNF1A-MODY groups. The amount of* Parabacteroides*,* unnamed genus of *Clostridiaceae,* Turicibacter*,* Lachnospira*, and* Anaerostipes* was higher, and the* unnamed genus of *Enterobacteriaceae was lower in the first control group than in T2DM group. The frequency of the* other genus of *Clostridiaceae and* SMB53* was lower in T2DM group than in the first control group and HNF1A-MODY group (supplementary data).

Comparing the microbiome between T2DM group and the second control group (matched with respect to age) revealed the differences in the following OTUs: the frequency of Proteobacteria (phyla level), Actinomycetales (order level), the* other family of *Actinomycetales, Promicromonosporaceae (family level), the* other genus of the other family of *Actinomycetales,* Cellulosimicrobium*,* Bulleidia*, and* Eubacterium* (genus level) was higher and the frequency of Clostridiaceae, Ruminococcaceae (family level), and the* unnamed and the other genus of *Clostridiaceae and* SMB53* (genus level) was lower in T2DM group than in the second control group (supplementary data).

**Figure 4:**
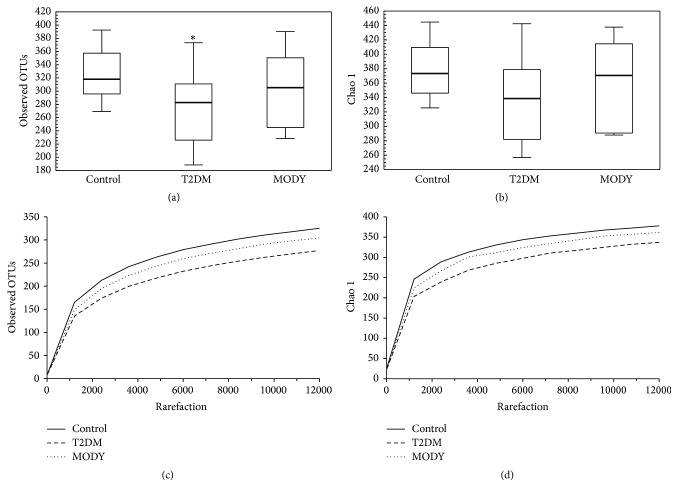
Box plots showing the alpha diversity ((a) observed OTUs, (b) Chao 1) comparison between groups. The rarefaction plots comparison ((c) observed OTUs, (d) Chao 1). Bold line: median; black lines: range of values. The observed OTUs but not Chao 1 alpha diversity were lower in T2DM group than in the control group. There were no significant differences in alpha diversity between T2DM and MODY and MODY and control groups. (a) Observed OTU alpha diversity: control versus MODY, *p* = 0.846; control versus T2DM, *p* = 0.027; MODY versus T2DM, *p* = 0.726. (b) Chao 1 alpha diversity: control versus MODY, *p* = 1; control versus T2DM, *p* = 0.063; MODY versus T2DM, *p* = 0.816. OTU: operational taxonomic unit; T2DM: type 2 diabetes mellitus; MODY: Maturity Onset Diabetes of the Young. *∗* indicates that there was a significant difference between this group and control group.

The observed OTUs alpha diversity (*p* = 0.027) but not Chao 1 (*p* = 0.063) was significantly lower in T2DM than in the control group; there was no difference in alpha diversity between control and HNF1A-MODY, HNF1A, and T2DM groups (Figure 4).

The beta diversity analysis revealed the differences in communities structure between T2DM and the second control group.

### 3.4. Continuous Glucose Monitoring

We obtained data of continuous glucose monitoring from 21 patients with T2DM and 9 patients with HNF1A-MODY. The mean glucose level in T2DM group was 167.33 ± 35.34 mg/dL, and the mean glucose level in HNF1A-MODY group was 143.11 ± 38.07 mg/dL; the groups did not differ in mean glucose level (*t*-test, *p* = 0.1037). The percentage of time with glucose level above 140 mg/dL was higher in T2DM group (Mann-Whitney *U* test, *p* = 0.0235); the percentage of time with glucose level within 70–140 mg/dL was higher in HNF1A-MODY group (Mann-Whitney *U* test; 29% (14%–54%) versus 49% (32%–81%), *p* = 0.0352); the percentage of time with glucose level below 70 mg/dL was higher in HNF1A-MODY group (Mann-Whitney *U* test; 2% (0%–6%) versus 0% (0%-0%), *p* = 0.0127).

## 4. Discussion

This study for the first time compares the bacterial flora of patients with HNF1A-MODY with the bacterial flora of individuals with T2DM and control group. Herein, we have shown for the first time significant differences in the bacteria profile of stool samples between HNF1A-MODY and both control and T2DM groups. It is true that the diabetes subpopulations differed at the baseline in age, duration of diabetes in years, BMI, and HbA1c. It is also possible that other confounders like diet [24] or medications [25–27] could influence the results. Some differences between the control group and diabetes cohorts can result from medications including antidiabetic drugs [26, 27]. It was demonstrated that the F/B ratio depends on age [28]. In infants and elders, it is significantly lower than in adults. It has also been presented that the obese people have increased F/B ratio [28].

T2DM and HNF1A-MODY groups in comparison with control group share some between-group differences but the study also revealed some type of diabetes-related differences. Between diabetes groups in OTU taxa, we observed that the frequency of Turicibacterales was significantly higher in HNF1A-MODY group than in T2DM group and in the first control group than in T2DM group. There were no differences between the first control and HNF1A-MODY groups. In the study results, the amount of Enterobacteriales was statistically higher in T2DM group than in the first control group. We cannot forget that only HNF1A-MODY group was matched with respect to age and BMI with the first control group. The baseline differences between T2DM and HNF1A-MODY group could be prone to and mask some differences in the microbiome profile between diabetes groups. Nevertheless, the study showed differences in the bacterial profile between HNF1A-MODY and control groups. This can be an argument for the thesis that also HNF1A-MODY is associated with changes in colonic bacterial flora.

The differences in microbiome profiles between the first control group and T2DM group were not the same as between the second control group and T2DM group. This could result from the differences in age; only the second control group was matched with respect to age with the T2DM group. Still, the second control group was not matched with respect to BMI with the T2DM group.

In recent studies, it has been revealed that the alpha diversity (within samples diversity) is decreased in obese individuals [29], but no significant differences were found in alpha diversity between healthy people and diabetes patients [30, 31]. In our study, we observed lower OTUs alpha diversity in T2DM group than in the control group, but the groups differ in BMI. We did not observe differences in alpha diversity between HNF1A-MODY and both control and T2DM groups. Nevertheless, the alpha diversity in HNF1A-MODY group was lower than in the control group and higher than in the T2DM group, but the differences were not significant.

One could suggest that the colonic microbiome plays a different role in the metabolic control in T2DM in comparison to HNF1A-MODY. This finding could have significant clinical implication. One could speculate that manipulation in the structure of gut microbiota, in order to improve glucose metabolism in patients with diabetes, could be more effective in some types of diabetes (like T2DM) than in others and should be individualized to the type or subtype of diabetes.

Some bacteria that belong to the Firmicutes phylum are butyrate producers. They play a key role in human gut energy supply by producing butyrate, the main source of energy for the colonic epithelium. Furthermore, butyrate improves insulin sensitivity [32] and is able to trigger the secretion of GLP-1 from L-cells [33]. Tolhurst et al. demonstrated that short fatty acids stimulate the secretion of GLP-1 in vitro [34]. Also, some suggest that there is a relationship between the short fatty acid as histone deacetylases inhibitors and the role of epigenetics in the development of diabetes [35]. It has to be emphasized that, in recent studies, it is reported that the amount of butyrate producers such as* Roseburia* species and* Faecalibacterium prausnitzii* is lower in patients affected with T2DM [11, 36]. In our data, the frequency of the genus* Faecalibacterium* was 0.5% in control, 0.2% in HNF1A-MODY, and 0.5% in T2DM group but the differences did not approach a level of significance (*p* > 0.05). The genus* Roseburia* was abundant in 0.5% in control, in 1.1% in HNF1A-MODY, and in 0.3% in T2DM (*p* > 0.05 for each comparison).

In our study, the relative abundance of* Akkermansia* tended to be higher in the first control group (6.5%) than in HNF1A-MODY (1.8%) and T2DM (3.9%) groups. Nonetheless, statistical significance was not reached (*p* > 0.05). It has been shown that in humans the percentage of* Akkermansia muciniphila* in the healthy population is 1–4% [37, 38]. The abundance of the bacteria was reported to be decreased in genetically (T2DM) and diet-induced obesity mice model [39]. On the other hand, it has been shown that in T2DM microbiota genes belonging to* Akkermansia *are increased [36]. Of note, four individuals in the T2DM group had very high relative abundance of* Akkermansia*: 31.4%, 32.1%, 21.1%, and 20.6%. Three controls from the first control group (29.2%, 17.8%, and 14.3%) and two HNF1A-MODY patients (9.5% and 13.7%) were also characterized by high frequency of* Akkermansia*. It was demonstrated that higher amount of* Akkermansia muciniphila* in overweight and obese people resulted in healthier metabolic status and better glucose homeostasis after introducing calorie restriction diet [40].

Definitely, one of the limitations of our study is due to the age differences between the T2DM and MODY individuals. The difference is due to the fact that MODY individuals are usually diagnosed at much younger age in comparison to patients with type 2 diabetes. The individuals from older generations representing MODY families were usually not available for genetic testing (either death or no willingness for testing). On the other hand, hardly ever do we diagnose type 2 diabetes in patients below 50 years of age. This is why matching T2DM and MODY patients for the age was not possible. To address these differences, we used two separate control groups. The first one was matched with MODY individuals with respect to age and BMI. The second one was matched with T2DM patients with respect to age. We failed, however, to match controls with T2DM as far as BMI is concerned (nondiabetic obese individuals were not willing to follow the study protocol).

Another limitation was related to the way we assessed dietary profiles of our patients. The estimation was based on a self-reported questionnaire and was not verified; thus, we cannot fully exclude the notion that the results of our study to some degree were affected by differences in dietary patterns.

However, the major limitation of our study was the relatively small sample size, which was partially due to protocol requirements. Follow-up studies based on larger groups are required to confirm the findings.

Comparison of bacterial profiles between patients with HNF1A-MODY and individuals with mutation in HNF1A gene without clinical diabetes could be significant.

It could be suggested that all of the particular genus of bacteria, proportion of bacteria (including shifts and the proportion within the same phyla, class, order, or family), and percentage of bacteria with the same function should be taken into account in consideration of the influence of microbiota on the development of different types of diabetes.

The differences in microbiota between patients with different types of diabetes could be also a novel strategy in distinguishing subtypes of diabetes.

In conclusion, it appears that there are differences in gut microbiome composition between patients with HNF1A-MODY, control individuals, and patients with type 2 diabetes. The clinical importance of this finding remains to be explored.

## Supplementary Material

The more detail information about the results of comparison of OTUs across samples and patients' treatment can be found in the supplementary data.
