# Supplementary material for: Qualitative Parameters of the Colonic Flora in Patients with HNF1A-MODY Are Different from Those Observed in Type 2 Diabetes Mellitus
Source: J Diabetes Res. 2016 Oct 11;2016:3876764. doi: 10.1155/2016/3876764 (PMC5078663; doi:10.1155/2016/3876764)

1. **The patients` treatment.**

| Type 2 diabetes group (23 patients) | HNF1A-MODY group (10 patients) |
| --- | --- |
| Metformin (MET) 12/23  MET + sulfonylurea (SU) 8/23  MET + SU + acarbose 1/23  SU: 2/23 | Insulinotherapy 5/10  Insulinotherapy + SU 1/10  SU 3/10  MET + SU 1/10 |

1. **The comparison of frequency of OTUs (operational taxonomic units) between groups (the first control group, HNF1A-MODY and T2DM group) at the class and order level.**

Class level

**
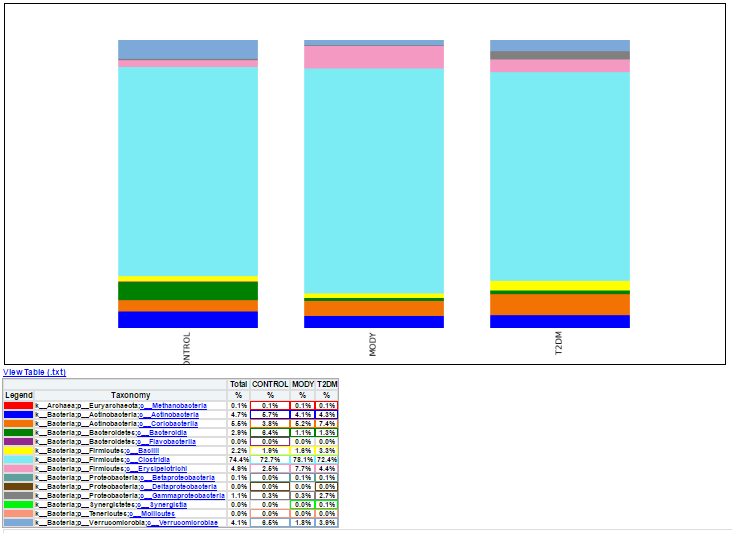
**

Order level


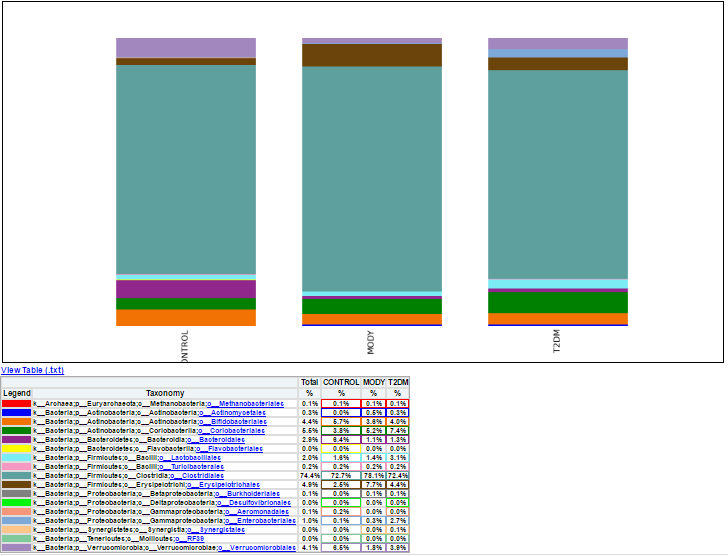


1. The comparison of frequency of OTU across sample groups (the first control group, HNF1A-MODY and T2DM group). The non-parametric ANOVA (Kruskal-Wallis test) was performed. When significant differences between groups were revealed the post-hoc tests were performed.


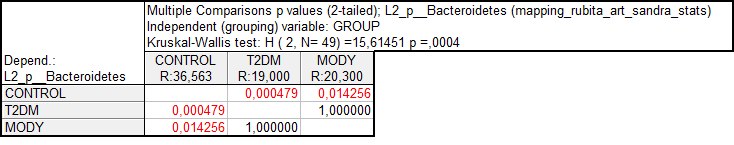


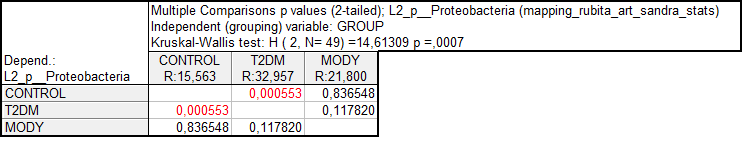


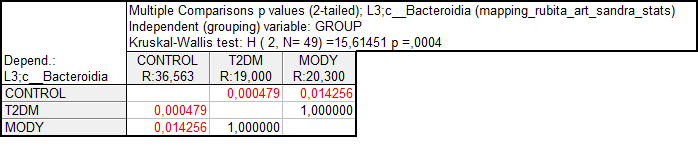


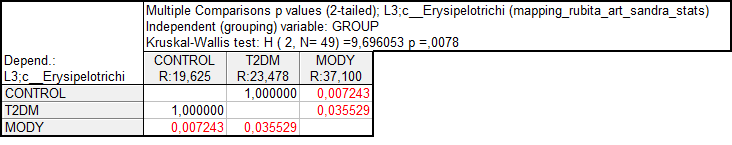


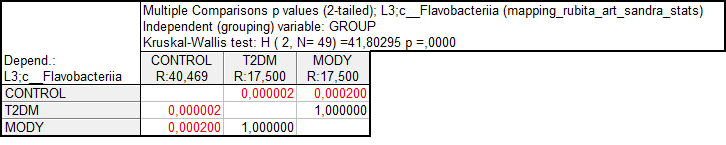


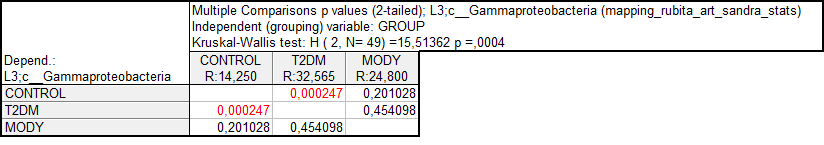


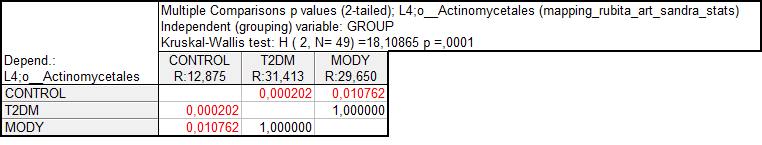


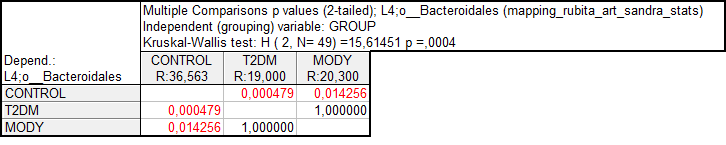


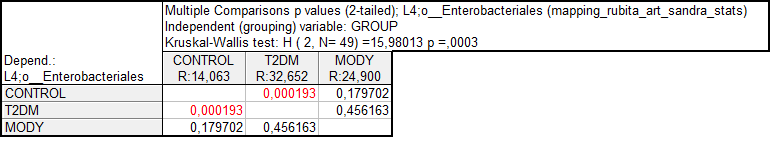


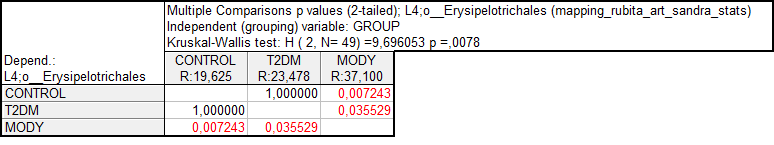


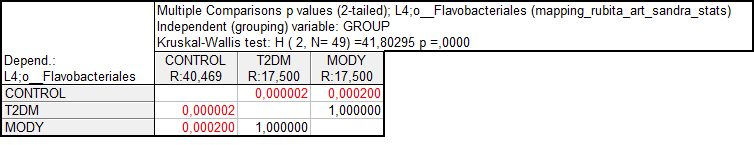


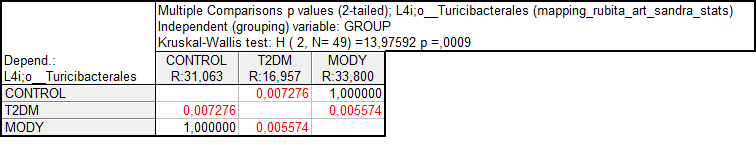


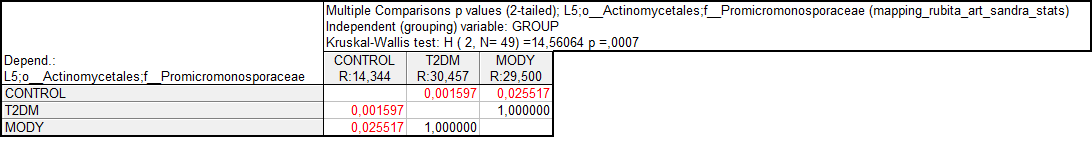


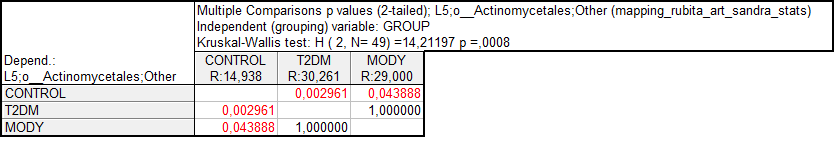


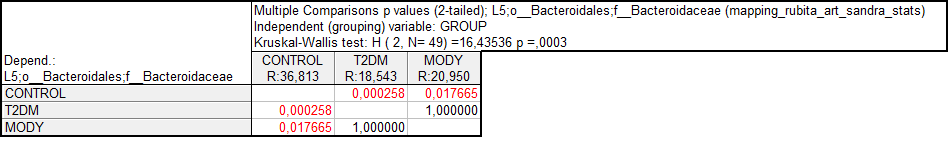


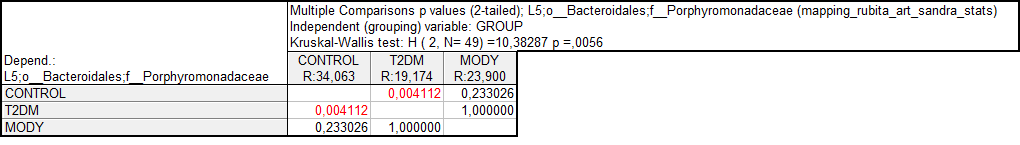


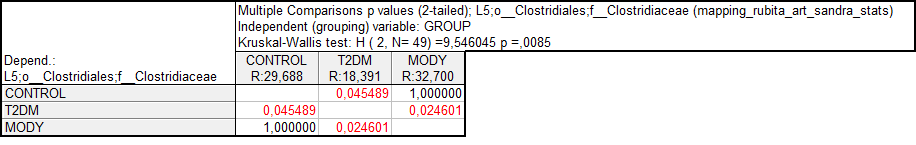

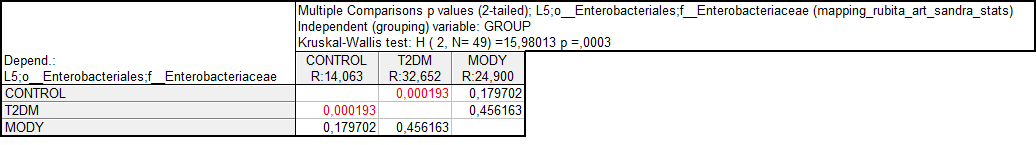


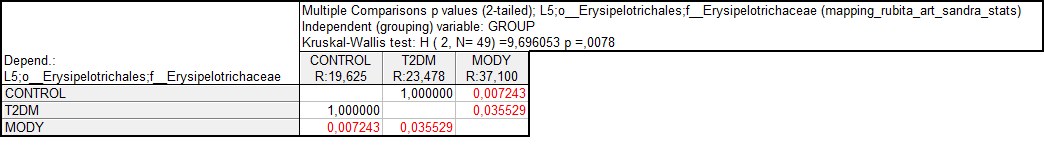


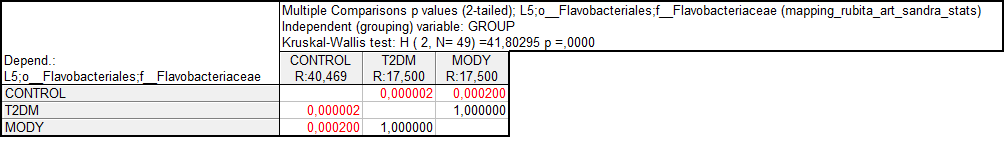


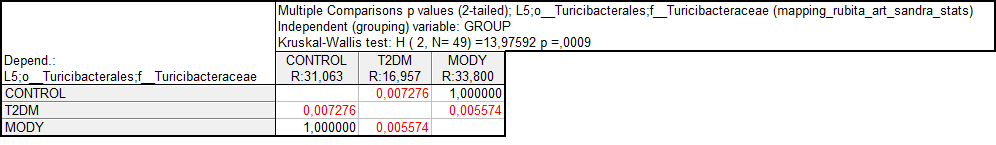


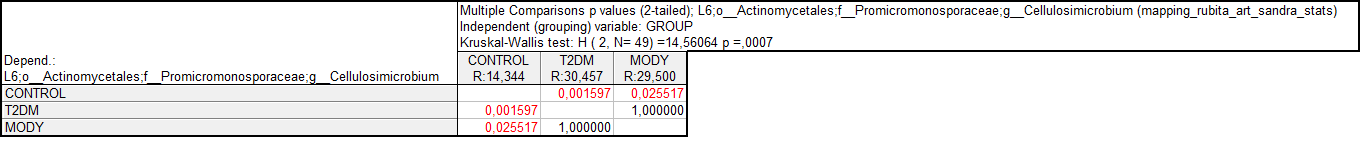


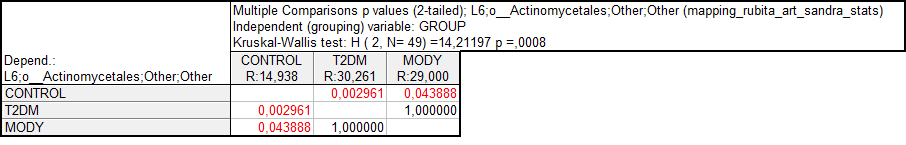


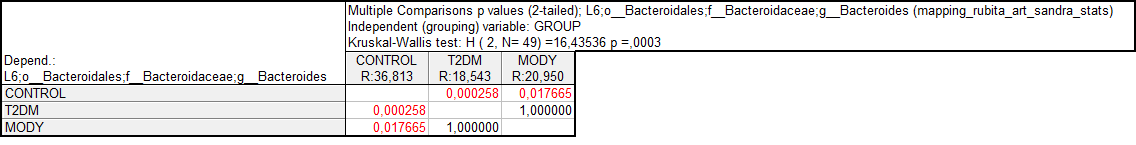


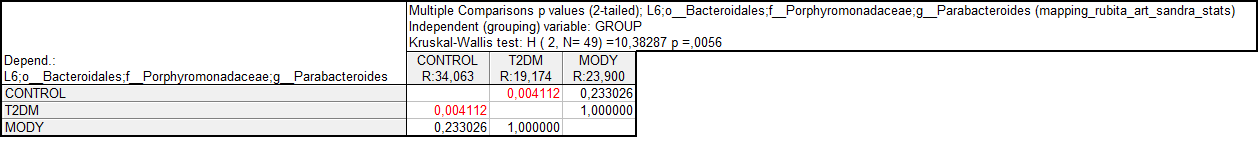


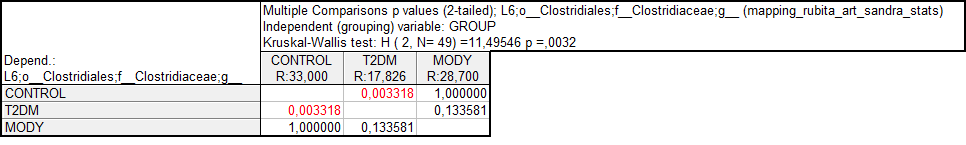


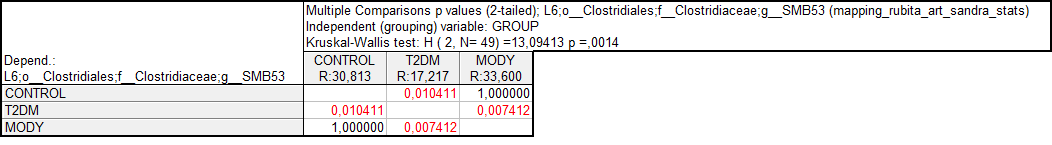


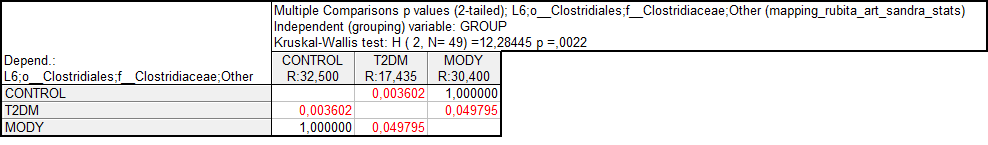


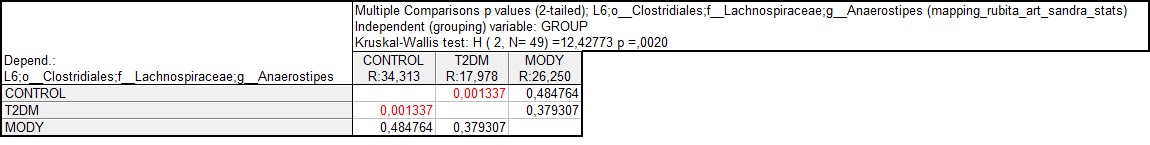


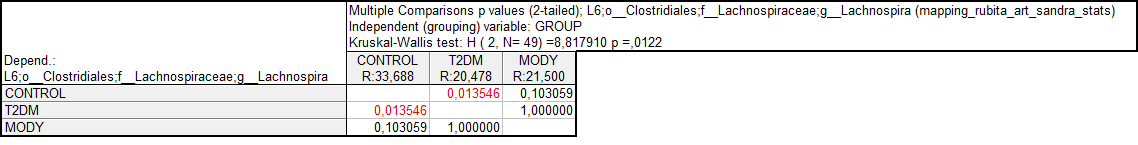


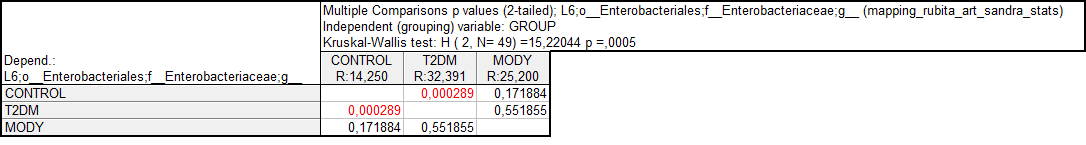


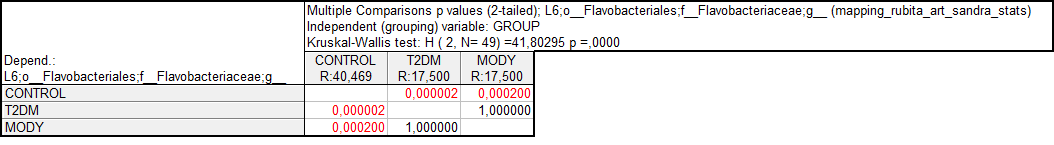


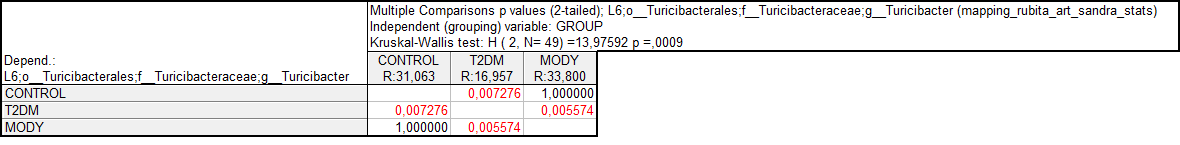


1. The comparison of frequency of OUT between T2DM group and the second control group. The Mann-Whitney test was performed.


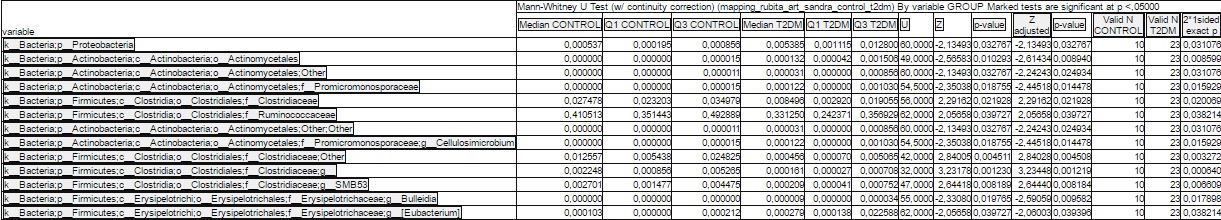

Supplement: Supplementary file 1 — The more detail information about the results of comparison of OTUs across samples and patients' treatment can be found in the supplementary data. [file 3876764.f1.docx]
